# Ultrafast Intermolecular Dynamics of Nanoconfined Water in Swollen Lipid Cubic Mesophases

**DOI:** 10.1002/smll.202508744

**Published:** 2025-10-03

**Authors:** Eva Zunzunegui‐Bru, Serena Rosa Alfarano, Patrick Züblin, Laura Baraldi, Hendrik Vondracek, Federica Piccirilli, Lisa Vaccari, Raffaele Mezzenga

**Affiliations:** ^1^ Department of Health Sciences and Technology ETH Zurich Zurich 8092 Switzerland; ^2^ Elettra Sincrotrone Trieste Strada Statale 14 km 163.5 in Area Science Park Basovizza Trieste 34149 Italy; ^3^ Diamond Light Source MIRIAM Infrared Beamline B22. Harwell Science and Innovation Campus Didcot OX11 0DE United Kingdom; ^4^ AREA Science Park Padriciano 99 Trieste 34149 Italy; ^5^ Department of Materials ETH Zurich Zurich 8092 Switzerland

**Keywords:** atomistic molecular dynamics, bicontinuous cubic mesophase, confined water dynamics, hydrogen bond network, intermolecular modes of water, re‐entrance, Terahertz absorption spectroscopy

## Abstract

Understanding the structure and dynamics of the hydrogen‐bond network of water in topologically distinct swollen lipidic mesophases, is fundamental for their application in biomedical, pharmaceutical, and food science fields. Here, a positive and non‐linear correlation between water hydrogen‐bond dynamics and interfacial water population is uncovered in inverse bicontinuous swollen mesophases across an extended temperature range (298–340 K). Particularly, small‐angle X‐ray scattering determines the mesophase's structural features, uncovering a temperature‐driven re‐entrant phenomenon (reappearance) of $Pn\bar{3}m$ phase upon heating. This topologically rich environment, however, has no detectable impact on the temperature dependence of the intermolecular modes of water, as revealed by terahertz absorption spectroscopy. Specifically, these modes show distinct dynamics: the stretching mode exhibits longer lifetimes than the libration mode, yet with a higher temperature‐dependence, with approximately two‐fold lower Arrhenius activation energies. In contrast, both stretching and libration modes exhibit a monotonic decrease in lifetime with increasing temperature, due to the increasing disruption of the hydrogen‐bond network. Atomistic molecular dynamics simulations enable the quantification of interfacial water population, which shows a positive correlation with intermolecular lifetimes in a nonlinear manner, revealing a non‐additive coupling between interfacial water population and water hydrogen‐bond network dynamics within these systems.

## Graphical abstract

Terahertz absorption spectroscopy reveals the temperature‐dependent intermolecular dynamics and structure of water in topologically distinct bicontinuous cubic lipid mesophases. Atomistic simulations show increased hydrogen‐bond disruption with heating and enable quantification of interfacial water, which correlates positively and nonlinearly with hydrogen‐bond lifetimes.

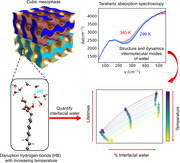

## 1 Introduction

Lipid inverse bicontinuous cubic phases (IBCPs) are versatile nanostructures that present unique opportunities in a broad range of applications, including drug delivery,^[^ ^1^, ^2^ ^]^ membrane protein crystallization^[^ ^3^, ^4^, ^5^ ^]^ and nanoreactors design.^[^ ^6^ ^]^ One of their key strengths is the increase in their interfacial surface area compared to hexagonal, micellar, or lamellar lipidic mesophases. Near this water‐lipid interface, water—which is confined within the water channels of the IBCPs— experiences entropic penalties, disrupting its hydrogen‐bond (HB) network. This disruption alters both the structure and dynamics of water, resulting in properties that deviate significantly from those of water's bulk phase.^[^ ^7^, ^8^ ^]^ The local tetrahedral ordering of the HB network is governed by the intermolecular vibrational modes of the water molecules^[^ ^9^ ^]^ and gives rise to its unique thermodynamic anomalies and exotic phase behavior.^[^ ^10^, ^11^ ^]^ Therefore, characterizing the intermolecular modes of water is fundamental for optimizing applications where interfacial water is present. Specifically, within lipid swollen IBCPs, and building upon a previous study where universal signatures of the HB network of nanoconfined water at room temperature were identified,^[^ ^12^ ^]^ this study pushes forward our understanding of the dynamic of the HB network in nanoconfined water and in particular, how this is influenced by soft nanoconfining interfaces. Particularly, we investigate the correlation between the dynamics of the intermolecular modes of water and the interfacial water population across biological and physiologically relevant temperatures (298–340 K). This study deepens our fundamental understanding of water HB network within these systems and advances the potential for their concrete applications in bio‐nanotechnology, pharmaceutical and food applications.

Specifically, the intermolecular vibrational modes of water arise from interactions between water molecules via HB and/or dipole‐dipole interaction. These modes are excited in the low‐frequency regime (10–1000cm^−1^), at the picosecond time scale, and are composed of hindered translations, rotations and bending motions.^[^ ^13^ ^]^ At high frequencies around 500 cm^−1^ (≈15.1 ps) the strong orientation correlations within water lead to strongly hindered rotations,^[^ ^13^ ^]^ also denominated as the librational intermolecular mode of water. At 180 cm^−1^ (≈5.4 ps) the hindered translation of water molecules presents an absorption feature which essentially is a hydrogen bond stretching vibration (stretching mode).^[^ ^14^ ^]^ Finally, below 50 cm^−1^ (≈1.5 ps) we can find the Debye relaxation related to the O─H⋅⋅⋅O bending.^[^ ^15^ ^]^

Intermolecular properties of the HB network in water can be indirectly probed via the OH stretching located in the infrared spectrum (>3000 cm^−1^).^[^ ^16^ ^]^ Techniques such as vibrational echo,^[^ ^17^, ^18^ ^]^ 2D pump‐probe^[^ ^19^, ^20^ ^]^ or optical Kerr effect^[^ ^21^ ^]^ provide valuable insight into ultrafast vibrational dynamics of water. At lower frequencies, Raman spectroscopy unveils features such as the O─H⋅⋅⋅O bending mode,^[^ ^22^, ^23^ ^]^ while dielectric spectroscopy can resolve the intermolecular stretching mode as well.^[^ ^24^ ^]^

**Figure 1:**
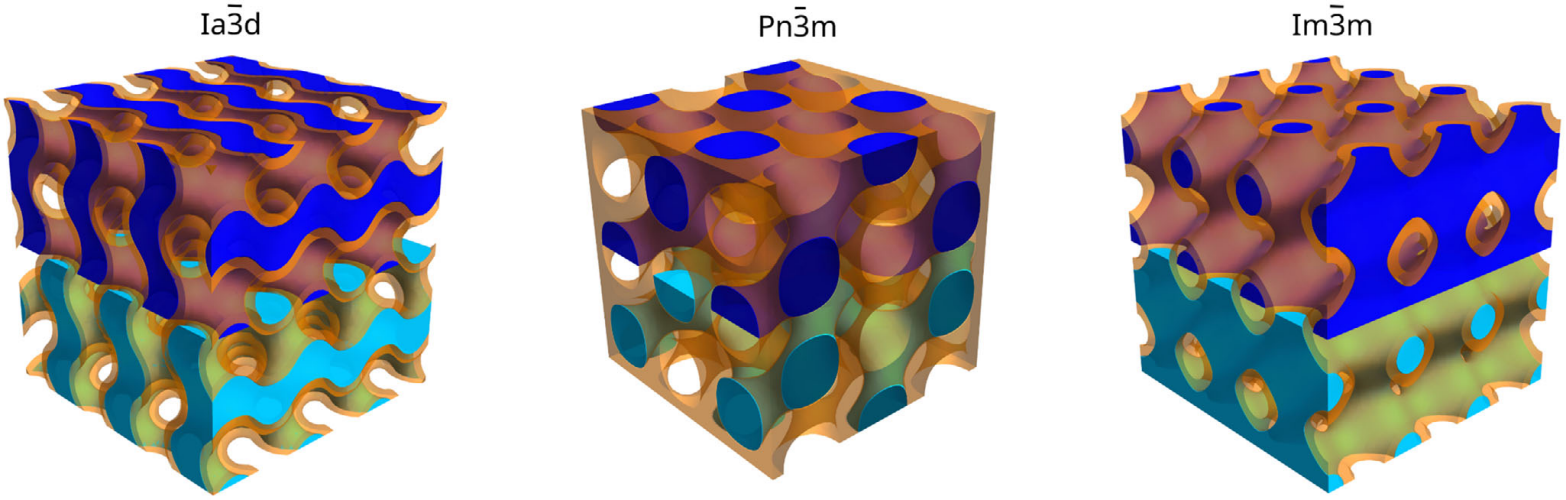
Schematic representation of repeating unit cells of the inverse bicontinuous cubic phases, described by the space groups $Ia\bar{3}d,\;Pn\bar{3}m,$ and $Im\bar{3}m$. The lipid bilayer is shown in orange, while the two interpenetrating but independent water channels are differentiated in light and dark blue.

In contrast, terahertz (THz) spectroscopy further extends access to the low‐frequency regime (30–500 cm‐1), enabling the resolution of both hindered translational, rotational and O─H⋅⋅⋅O bending motions of water molecules. When combined with simulations, THz data can be interpreted to extract the microscopic mechanisms underlying the HB network of water.^[^ ^25^ ^]^ For instance, it enables the characterization of the undercoordination of interfacial water within clathrates hydrates^[^ ^26^ ^]^ or hydrophobic hydration shells.^[^ ^27^ ^]^ Within topologically distinct IBCPs, this combination has uncovered the universal features of the structure and dynamics of nanoconfined water in these systems at room temperature.^[^ ^12^ ^]^ The characterization of the HB network of water within IBCPs is, however, not trivial. For instance, the population of interfacial water was found to be larger in IBCPs compared to the hexagonal phase, as determined by broadband spectroscopy (<1 cm^−1^)^[^ ^28^ ^]^ or their water dynamics were revealed to be faster than those within reverse micelles via 2D infrared spectroscopy.^[^ ^29^ ^]^ Particularly, in contrast to the hexagonal phase or reverse micelles, IBCPs describe infinite triply periodic minimal surfaces^[^ ^30^ ^]^ within which two set of interconnected but independent and non‐communicating water channels can be identified^[^ ^31^ ^]^(**Figure** **1**). Notable examples include, gyroid,^[^ ^32^ ^]^ diamond (D) and primitive (P) surfaces,^[^ ^33^ ^]^ characterized by the space groups $Ia\bar{3}d,\;Pn\bar{3}m$ and $Im\bar{3}m$, respectively (Figure 1). In these structures, the ubiquity of the lipid membrane (orange region in Figure 1) partitions the space into two identical, non‐communicating, and interpenetrating water channel networks, shown in dark and light blue in the schematic representation in Figure 1. Here, we build upon the previous findings at room temperature within swollen IBCPs^[^ ^12^ ^]^ to investigate how temperature and interfacial water shape the HB network. In particular, we address the open question of how the interplay between interfacial water, topology, and temperature govern the intermolecular vibrational modes of water.

To tackle this conundrum, we conduct a systematic investigation of the temperature dependence (298–340 K) of the intermolecular modes of water, probed via THz absorption spectroscopy, within topologically rich swollen lipid mesophases. Specifically, we incorporate a co‐surfactant, i.e., sugar ester (SE), to dimodan‐water systems, with dimodan being the commercial counterpart of monolinolein monoglyceride,^[^ ^34^ ^]^ to facilitate the stabilization of mesophases with the three distinct IBCPs at hydration levels of 30%, 40%, and 50%. Small‐angle X‐ray scattering (SAXS), underscores the critical role of the SE in modulating the temperature‐dependent phase behavior of dimodan‐water systems, uncovering a temperature‐driven re‐entrant phenomenon in the most hydrated system. THz absorption spectroscopy reveals that stretching mode dynamics are slower and more temperature‐sensitive than liberation. Despite these differences, both modes display a common trend: increasing confinement leads to longer vibrational lifetimes. Atomistic MD simulations offer molecular‐level insight into this behavior, revealing temperature‐invariant distance from the membrane bilayer center until which interfacial water is detected across systems. This enables the analytical quantification of interfacial water, revealing a positive and nonlinear correlation with vibrational lifetimes. Together, these findings underscore the profound and nontrivial influence of soft nanoconfinement on the temperature‐dependent structure and dynamics of the HB network.

## 2 Results and Discussion

### 2.1 SAXS Reveals Nontrivial Temperature‐Driven Phase Behavior of Dimodan‐Water Swollen Mesophases

Pure dimodan‐water systems reach a maximum swelling capacity at ≈35% hydration,^[^ ^35^ ^]^ SE co‐surfactant helps overcome this limitation,^[^ ^34^ ^]^ enabling a controlled and tunable swelling, which is essential for applications such as membrane protein crystallization^[^ ^4^ ^]^ and tunable diffusion of solutes.^[^ ^34^, ^36^ ^]^ In this study, we achieve hydration of the mixture SE‐dimodan of 30% (SE10‐W30), 40% (SE10‐W40) with 10 wt.% SE, and 50% hydration (SE20‐W50) with 20 wt.% SE content, where SE content is quantified relative to the total lipid mixture (SE + dimodan). Particularly, IBCPs present in these swollen mesophases exhibit three‐dimensional long‐range translational order, which gives rise to Bragg reflections detectable by SAXS^[^ ^37^ ^]^ and allows us to characterize their temperature‐dependent structure (see Experimental Section).

**Figure 2:**
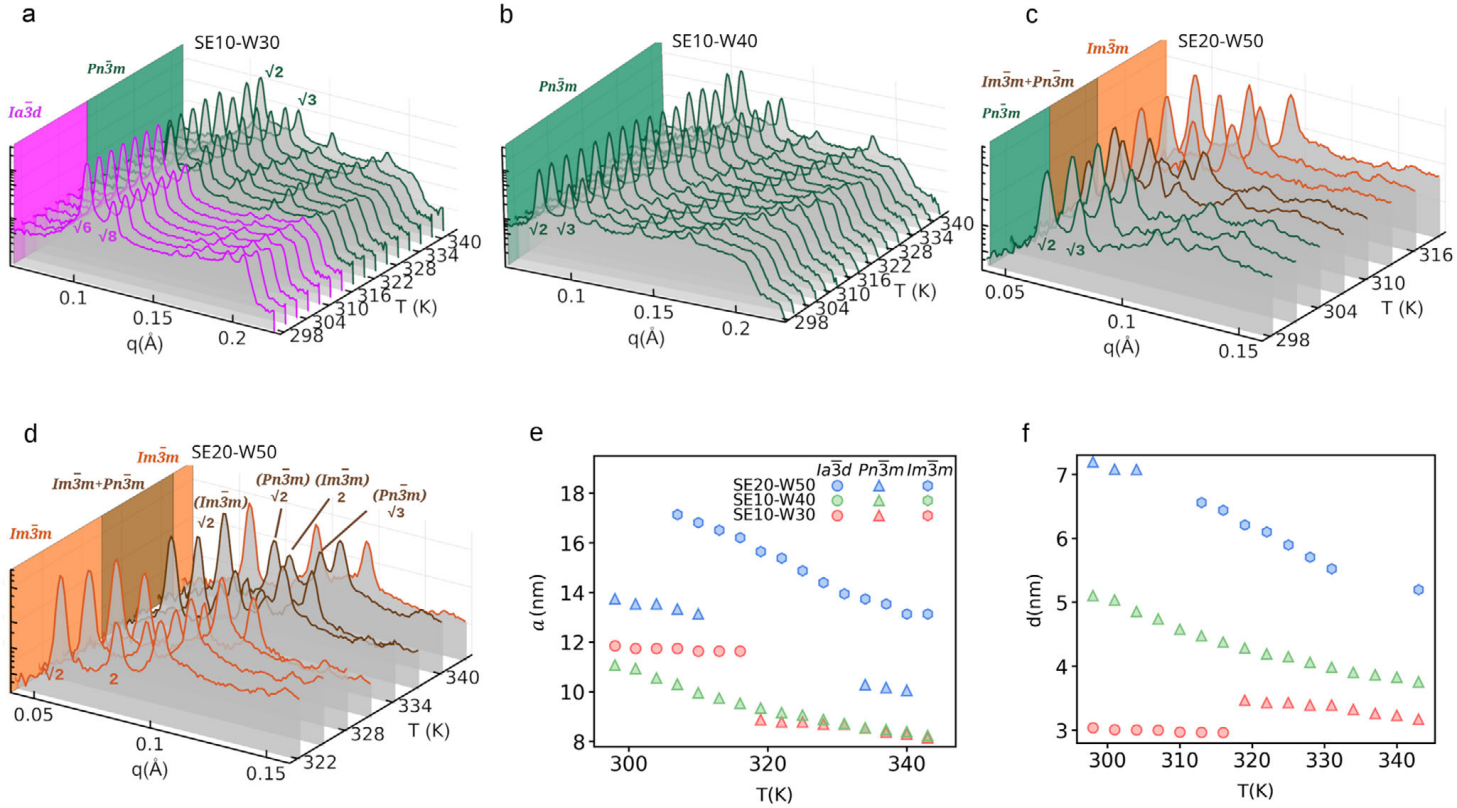
SAXS structural temperature‐dependent characterization. a–d) SAXS profiles for SE10‐W30, SE10‐W40, and SE20‐W50 samples across the temperature range of 298–343 K. For clarity, the SAXS profiles of SE20‐W50 sample are split into two panels: c) 298–319 K and d) 322–343 K. The identified phases are differentiated by color. In all panels, the first two Bragg reflections of the representative IBCPs are indicated: $\left\{\sqrt{6},\sqrt{8}\right\}$, {$\sqrt{2},\sqrt{3}\},\;\left\{\sqrt{2},2\right\}$ for $Ia\bar{3}d,\;Pn\bar{3}m$ and $Im\bar{3}m$ respectively. e) Lattice parameter (*a*) as a function of temperature for all investigated systems. f) Average water channel diameter (*d*) as a function of temperature, calculated only for pure phases; phase mixtures are excluded due to the inability to experimentally resolve the water distribution between coexisting phases. In panels e and f, the phases and samples are distinguished using both color and symbol coding respectively.

Specifically, in the sample SE10‐W30, we observe a thermally driven transition $Ia\bar{3}d\to Pn\bar{3}m$ at T = 316 K (**Figure** **2**a). This transition is energetically favorable and is accompanied by a reduction in lattice parameter and an increase in the diameter of the water channels (Figure 2c,d). The temperature‐driven transition arises from two main effects: (i) the progressive disruption of hydrogen bonds (HBs) between water molecules and lipid headgroups at elevated temperatures, which reduces headgroup hydration^[^ ^38^, ^39^ ^]^; and (ii) the increase in thermal motion of the hydrocarbon tails, which enhances packing frustration within the monolayer.^[^ ^40^ ^]^ An equivalent isothermal transition is also induced by increasing hydration at room temperature, as seen when comparing SE10W30 to SE10W40 (Figure 2a,b). The $Ia\bar{3}d$ phase is known to be thermodynamically favored at lower hydration levels and is characterized by larger lattice parameters relative to the $Pn\bar{3}m$ phase^[^ ^35^ ^]^ (Figure 2e). This increase in hydration also results in wider aqueous channels, as observed in this $Pn\bar{3}m$ in SE10‐W40 as compared to the $Ia\bar{3}d$ in SE10‐W30 at room temperature (Figure 2f, see Experimental Section for more information on the calculation of the average channel diameter of the IBCPs). Overall, we can conclude that $Ia\bar{3}d\to Pn\bar{3}m$ phase transition, whether driven by temperature or hydration, is associated with a decrease in the water‐lipid HBs. This suggestion is rooted in the approximately 1.6 relative decrease in the dimensionless specific surface area of the $Pn\bar{3}m$ as compared to the $Ia\bar{3}d,$ ^[^ ^41^ ^]^ which leads to a reduced number of water molecules in contact with the polar groups of dimodan. This transition should therefore be entropically favored since water reduces the interaction with the water‐lipid interface. In other words, this reduction mitigates the entropic penalties associated with water interacting with interfaces.

Furthermore, as previously reported^[^ ^12^, ^34^ ^]^ the inclusion of SE enables the formation of the primitive inverse bicontinuous cubic phase in dimodan‐water systems at higher SE concentrations. In the sample SE20‐W50, increasing the temperature induces the emergence of the $Im\bar{3}m$ phase in coexistence with $Pn\bar{3}m$ at *T* = 307 K (Figure 2c). Upon further disruption of HBs with continued temperature increase, the $Im\bar{3}m$ becomes dominant, replacing $Pn\bar{3}m$ entirely. This transition is accompanied by a significant increase in both the lattice parameter and the diameter of the water channels (Figure 2e,f).

Strikingly, at 334 K in the sample SE20‐W50, we observe a re‐entrant appearance of the $Pn\bar{3}m$ phase upon heating, coexisting with the previously dominant $Im\bar{3}m$ phase (Figure 2d). This phenomenon, i.e the re‐entry of $Pn\bar{3}m$ from an $Im\bar{3}m$ phase, has previously been reported in charged phospholipid systems such as monopalmitolein mesophases upon increasing hydration.^[^ ^4^ ^]^ To the best of our knowledge, this is the first time such re‐entrant behavior is observed as a function of temperature.

We interpret this first‐order transition in terms of the thermodynamic stabilization of curvature energy at a critical lattice parameter. Notably, the lattice parameter of the $Im\bar{3}m$ phase decreases with increasing temperature and reaches values comparable to those of $Pn\bar{3}m$ at lower temperature, precisely at the temperature where re‐entry occurs. This suggests that at this lattice size, the curvature energy becomes thermodynamically favorable for both $Im\bar{3}m$ and $Pn\bar{3}m$, enabling the reappearance of the $Pn\bar{3}m$ phase. The coexistence of these two phases results in a decrease in lattice parameter of $Pn\bar{3}m$ compared to that observed at lower temperatures.

Intriguingly, the $Ia\bar{3}d$ lattice parameter shows little to no dependence on temperature (Figure 2e), in contrast to the monotonic decrease observed for both $Pn\bar{3}m$ and $Im\bar{3}m$. This finding indicates that $Ia\bar{3}d$ is the most tightly packed phase, i.e., it incorporates less hydration water, and is therefore less sensitive to HB disruption with increasing temperature. This is consistent with previous differential geometry‐based analyses reported in the literature.^[^ ^42^ ^]^

As a final note, we find that the presence of SE induces significant modifications in the temperature‐dependent phase behavior of the dimodan‐water system. First, the $Pn\bar{3}m$ phase is stabilized upon heating (above ≈ 328 K) in the system SE10‐W40, in contrast with pure dimodan‐water systems, where this phase underwent a transition to the highly curved hexagonal (*H_II_*) phase.^[^ ^35^ ^]^ Second, at higher SE content (sample SE20W50), the $Im\bar{3}m$ phase emerges in the most hydrated system. The lattice parameter of this phase decreases monotonically with increasing temperature, reaching values at which $Pn\bar{3}m$ was previously found to be thermodynamically stable at lower temperatures. This leads to a re‐entrant appearance of the $Pn\bar{3}m$ IBCP.

Taken together, these findings highlight the critical role of SE as a co‐surfactant in modulating lipid packing frustration, promoting the formation of swollen bicontinuous cubic structures, suppressing transitions to phases of higher curvature such the hexagonal phase and enabling temperature‐induced re‐entrant behavior.

### 2.2 THz Absorption Spectroscopy Resolves Temperature‐Dependent Fingerprints of Intermolecular Water Modes in IBCPs

**Figure 3:**
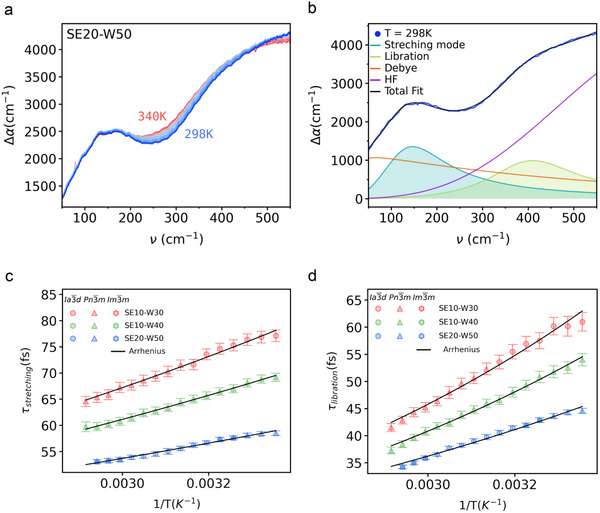
THz absorption spectroscopy characterization of the intermolecular vibrational modes of water. a) Water THz absorption spectra (Δα) for SE20‐W50 sample across the temperature range 298–340 K, measured in 3 K increments. b) Representative modelling using modified damped harmonic oscillators of Δα, for the SE20‐W50 sample at 298 K. The stretching and libration modes are shown with filled lines in turquois and green respectively, while low (Debye) and high (HF) frequency modes are indicated with orange and violet lines. Experimental data is presented with blue points and the total fit with a black line. c) Lifetime of the stretching mode (τ_*stretching*_) as a function of inverse temperature for all investigated samples. d) Lifetime of the libration mode (τ_*libration*_) as a function of inverse temperature for all investigated samples. In panels c and d samples are differentiated by color and the results are modelled with temperature‐dependent Arrhenius behavior (black line).

We then set to tackle the impact of nanoconfinement level uncovered by SAXS on the intermolecular structure and dynamics of water within the dimodan‐SE‐water systems using THz absorption spectroscopy in the low‐frequency range (50–550 cm^−1^). **Figure** **3**a presents the water spectra for the most hydrated sample (50 wt.%) across all the investigated temperatures from 298 to 340 K. The absorption coefficient (α) is obtained with the Beer‐Lambert law and Δα represent the spectra after subtraction of the lipid contribution (see Methods for more details). At first glance, no significant shift in the stretching band (≈180 cm^−1^) is observed in the spectra. This indicates that the rigidity of the network is not significantly affected in this temperature regime. In contrast, we discern a slight change in the width of the spectra in this region, pointing toward a change in the dynamics of the hindered translation. On the other hand, as there is spectral overlap between the librational mode (≈400 cm^−1^) and higher frequency contributions, an interpretation of spectral changes in this range is not straightforward. Nevertheless, our results indicate that increasing the temperature might result in a broadening of this intermolecular mode as well. The samples with less hydration (30 and 40 wt.%) show equivalent features (Figure S1, Supporting Information). A model mainly based on damped harmonic oscillators allow us to uncover the fingerprints on the temperature dependence of the investigated systems. Many studies have shown the relevance of this approach, which allows to disentangle the behavior of the intermolecular modes of water in THz absorption spectra.^[^ ^27^, ^43^ ^]^ Specifically, the model is defined as,

(1) $$\Delta \alpha \;\left(\nu \right)=a_{LF}\;\alpha_{LF}\left(\nu \right)+a_{HF}\alpha_{HF}\left(\nu \right)+\sum_{i=1}^{N}L_{i}\left(\nu \right)$$

where α_*LF*_(ν), α_*HF*_(ν) are the low and high contributions overlaping with our frequency range with their corresponding amplitudes *a_LF_*, *a_HF_*. The low frequency (LF) is assigned to Debye relaxation mode as observed by dielectric relaxation spectroscopy in resonances,^[^ ^44^ ^]^ while α_*HF*_ represents the overlapping high frequency terms, modelled with a modified damped harmonic oscillator (DHO) line (see Methods for more information). Finally, the intermolecular modes of water are described with,

(2) $$L_{i}\;\left(\nu \right)=\frac{a_{0,i}\omega_{0,i}^{2}\nu^{2}}{4\pi^{3}\left({\left(\nu_{0,i}^{2}-\nu^{2}\right)}^{2}+{\left(\frac{\omega_{0,i}}{2\pi }\right)}^{2}\nu^{2}\right)\;}$$

which is a slightly modified form of the DHO, where *a*_0,*i*_,  ν_0,*i*_,  ω_0,*i*_ are the amplitude, center frequency, and width of the two intermolecular modes of water, i.e., stretching and libration. Figure 3b presents all the aforementioned vibrational modes, for the water absorption spectra at 298 K in the sample SE20‐W50 (see Supporting Information for the complete set of investigated spectra, Figures S2‐S4, Tables S1‐S3, Supporting Information). The application of this framework allows us to quantify the rigidity of the network, as reflected by the center frequencies of the stretching and librational modes, which as mentioned above do not show significant shift across the investigated temperature range.

The temperature‐averaged center frequency of the stretching mode, ν_0,*stretching*_, are 144.2 ± 0.1, 145.2 ± 0.1 and 147.2 ± 0.1 cm^−1^, for SE10W30, SE10W40 and SE20W50 respectively. These values are considerably red shifted as compared to bulk water,^[^ ^25^ ^]^ attributed to the decrease in the coordination of the HB network resulting from presence of an interface, which disrupts the local interactions of water. This undercoordination is consistent with previous observations in systems involving hydrophobic hydration,^[^ ^27^ ^]^ water confined within clathrates cages^[^ ^26^ ^]^ and lipid mesophases.^[^ ^12^ ^]^ Specifically, these studies show that the stretching mode can be expressed in terms of two water populations: a bulk‐like component centered around 180 cm^−1^ and a red‐shifted contribution from interfacial water. In our study, we focus on the vibrational dynamics as reflected in subtle changes in the bandwidth of the stretching mode (Figure 3a). Given the intricacy of damped harmonic oscillator (DHO) modeling and the small magnitude of spectral width variations in the stretching frequency region, we analyze the spectrum as a single water population, thereby gaining insight into the overall impact of nanoconfinement on the dynamics of the stretching mode. Specifically, in,^[^ ^12^ ^]^ using equivalent swollen dimodan‐water systems, the two water populations were identified at ≈120 and 175 cm^−1^, corresponding to interfacial and bulk‐like water, respectively. These values align with our results, which show a center frequency located approximately midway between the two (≈147 cm^−1^), consistent with a superposition of both populations. The fingerprints from the existence of these two populations of water is unveiled by the consistent trend observed across ν_0,*stretching*_, which slightly increases monotonically (blue shift) with decreasing confinement, from channel diameters of ≈ 3 nm in SE10W30 to ≈ 7 nm in SE20W50 (Figure 2e). This blue shift reflects a decrease in the population of interfacial water as confinement is relaxed, indicating an overall increase in HB coordination, and consistent with a gradual increase in the bulk‐like population and a higher value of ν_0,*stretching*_.

On the other hand, the center frequency of the libration mode, ν_0,*libration*_, although centered around 415 cm^−1^, shows a slight red‐shift trend with decreasing confinement. The averaged values, with no significant impact on temperature, are 417.3 ± 0.3, 415.5 ± 0.2, and 409.9 ± 0.1 cm^−1^, for SE10‐W30, SE10‐W40, and SE20‐W50 respectively. This behavior suggests an anticorrelation between hindered translations and rotations: as translational motion becomes more restricted, librational rigidity decreases. We hypothesize that this behavior arises from a cooperative effect linked to the connectivity of the HB network.^[^ ^45^, ^46^ ^]^ In particular, the energetically favorable tetrahedral arrangement of water molecules leads to local structural confinement, wherein each molecule is effectively “caged”, by hydrogen‐bonded neighbors. This effect becomes more pronounced upon supercooling,^[^ ^47^ ^]^ however, within our investigated temperature range, such caging still occurs on ultrafast timescales (fs). Consequently, an increase in tetrahedral order, blue shift stretching mode, may enable greater rotational (librational) freedom, thereby explaining the observed red‐shift in the libration frequency.

At the same time, the dynamics reveal interesting features as the temperature increases. Specifically, we quantify the lifetime (τ_*i*_) of both intermolecular modes, which is related to the autocorrelation of the dipole moments, as inversely proportional to the width *w*_0,*i*_ via, τ_*i*_ =  1/*w*_0,*i*_ *c*, where *c* is the speed of light.^[^ ^23^, ^48^ ^]^

Figure 3c,d shows the lifetimes of the intermolecular modes of water associated with hindered translation and rotation, i.e., stretching (τ_*stretching*_) and libration (τ_*libration*_), plotted as a function of inverse temperature. All in all, increasing temperature results in faster relaxation dynamics, i.e., shorter lifetimes for both modes. The extracted lifetimes fall within the range reported in previous studies. In particular, lifetimes shorter than 100 fs have been reported via theoretical ultrafast studies for hindered rotations^[^ ^49^ ^]^ while THz absorption spectroscopy has revealed intermolecular modes of water have shown lifetimes in the range of 50–200 fs.^[^ ^43^ ^]^

In summary, τ_*streching*_ exhibits values larger than τ_*libration*_ within the same system and temperature. This trend is consistent with previous computational studies, which report approximately a two‐fold increase in HB relaxation times compared to rotational dipolar relaxation times.^[^ ^39^ ^]^ While these times scales are not directly comparable with the experimental intermolecular vibrational lifetimes, they nonetheless capture the quantitative distinction between collective stretching and librational dynamics.

Further analysis of both vibrational modes reveals an Arrhenius temperature dependence with activation energies shown in **Table** **1**. The results indicate that the stretching mode although having longer lifetimes, is more sensitive to temperature than the libration mode (Figure 3c,d). The obtained activation energy barriers of the HBs are below the bulk water value obtained previously via IR pump‐probe spectroscopy (14.21–15.476.3 kJ mol^−1^).^[^ ^44^, ^45^ ^]^ This change is in line with the lifetimes determined by these experiments, which are approximately an order of magnitude longer than those observed in our study, reaching values as high as 1 ps.^[^ ^45^ ^]^ These differences likely arise from the different frequency ranges probed by each method, which can yield distinct dynamical and energetical scenarios.

**Table 1:** Arrhenius activation energies (E_a_) in kJ mol^−1^ of the stretching and libration modes of the three investigated samples measured via THz Absorption spectroscopy.

	SE10‐W30	SE10‐W40	SE20‐W50
E_a_ Stretching mode	3.52	3.01	2.19
E_a_ Libration mode	7.44	6.77	5.27

Furthermore, the activation energies exhibit an inverse correlation with hydration (Table 1). A similar inverse trend is observed for the vibrational lifetimes. Specifically, Figure 3b,c unmistakably shows that both τ_*streching*_ and τ_*libration*_ decrease with increasing hydration across all samples. These findings collectively suggest that the amount of interfacial water plays a central role in modulating the intermolecular vibrational dynamics of confined water. In particular, reduced overall hydration leads to a relative increase in the interfacial water fraction within the system, thus amplifying its contribution to the observed dynamics. We explore this relationship in further detail in the following section using molecular dynamics simulations.

As a final observation, as seen in Figure 3c,d, the change in topology within the same sample has no discernible influence on the temperature‐dependent spectroscopic features of water's intermolecular dynamics within dimodan‐water swollen mesophases. Specifically, the lifetimes of both stretching and libration modes follow the same Arrhenius behavior after a phase transition, as exemplified by samples SE10‐W30 and SE20‐W50 (Figure 3c,d). This suggests that the universality previously observed for interfacial water at room temperature extends across a broader temperature range.

### 2.3 Interfacial Water and its Impact on the Hydrogen‐Bond Network Enabled by Temperature‐Dependent Atomistic Simulations

Experimental findings from SAXS and THz have revealed key aspects of the structure and dynamics of water confined within dimodan‐water swollen mesophases. However, the specific contribution of interfacial water to the overall water dynamics remains to be disentangled. With atomistic molecular dynamics simulations, we gain insight into the local mechanisms that govern the HB network of nanoconfined water. Previous investigations into topologically distinct swollen dimodan‐SE mesophases have found that, at room temperature, the structural and dynamical properties of the HB network of water for IBCPs and a hypothetical lamellar phase (with similar hydration and water channel dimensionality) evolve via the same universal functions of the distance from the dimodan‐water interface.^[^ ^12^ ^]^ In this work, we extend that analysis to explore the temperature dependence of the water HB network. Specifically, we focus on the same lamellar system as in that previous study,^[^ ^12^ ^]^ which is less computationally demanding. At the investigated hydrations, the lack of curved interfaces is not expected to have a discernible influence on the structure and dynamics of water. We simulate this system at increasing temperatures between 308 and 338 K with OPC water model (see Methods for further details). Specifically, we note that this is a non‐polarizable model which does not consider charge transfer and quantum effects. These effects influence the HB network by capturing the ability of water to dissociate into hydronium and hydroxide ions,^[^ ^50^, ^51^ ^]^ which is important for the chemical and biological role of water. In our case, however, at neutral pH, the concentration of these ions is low (10^−7^ m) and therefore is not anticipated to have a notable impact on the results.

**Figure 4:**
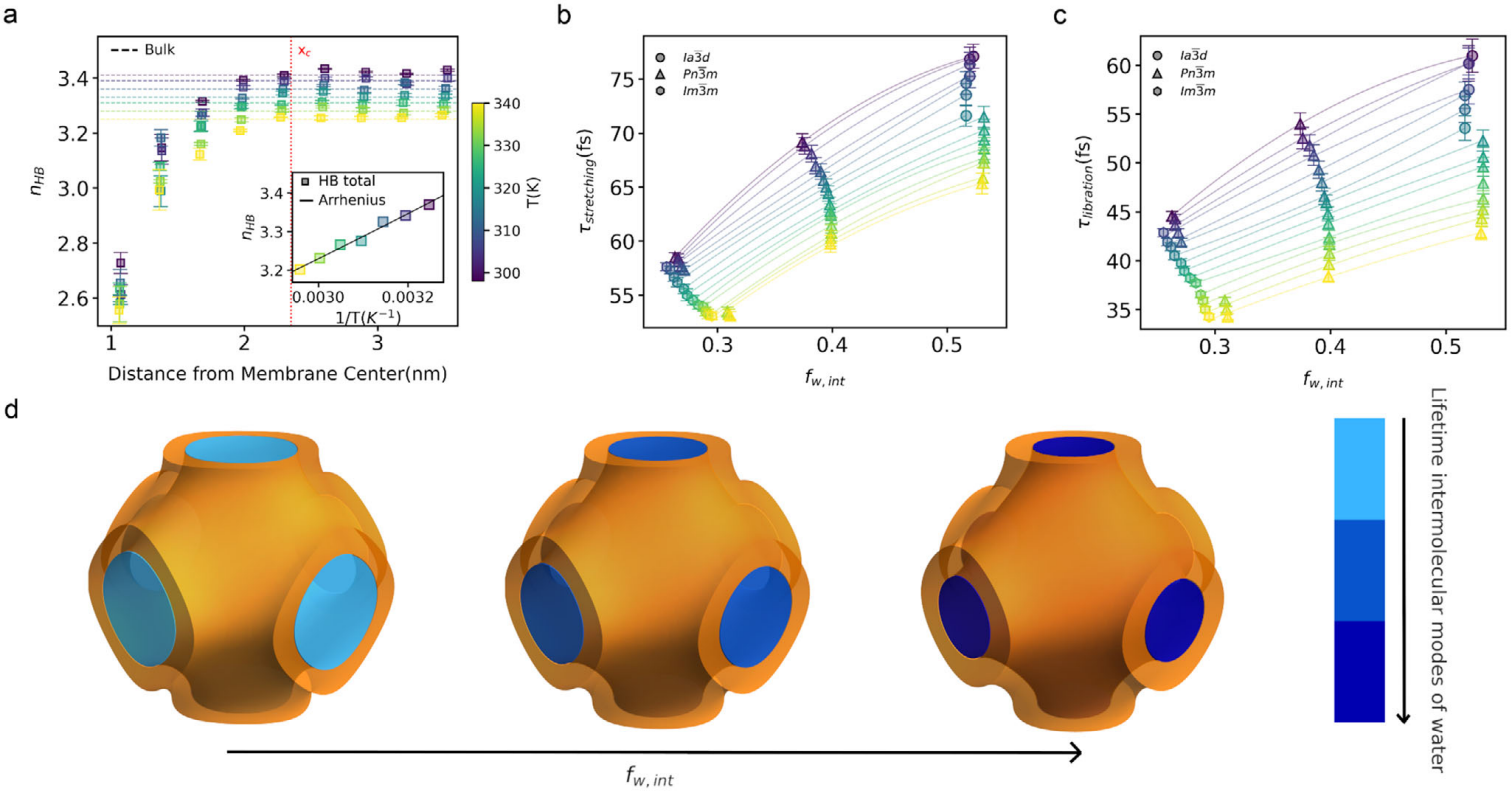
Interfacial water HB network within topologically distinct swollen mesophases. a) Average total number of HB per water molecule (*n_HB_*) as a function to the distance from the membrane center across the temperature range 308–338 K, where the bulk value at the investigated temperatures is indicated with horizontal dashed lines, *x_c_* indicates the distance at which the bulk *n_HB_* is recovered at all temperatures. Inset: *n_HB_* as a function of temperature modelled with Arrhenius temperature‐dependent behavior, yielding an activation energy of 1.48 kJ mol^−1^. b) Lifetime of the stretching mode (τ_*stretching*_) of water as a function of the interfacial water fraction (*f*_*w*,*int*_) across the temperature range 298–340 K. c) Lifetime of the libration mode (τ_*libration*_) of water as a function of the interfacial water fraction (*f*_*w*,*int*_) across the temperature range 298–340 K. In all panels the temperature is differentiated using a color gradient. In panels b and c, the lines correspond to non‐linear fits of the data. d) Schematic of the common picture obtained in our study on the dynamics of the intermolecular modes of water within IBCPs. The increase in interfacial water fraction (*f*_*w*,*int*_) is illustrated with three $Im\bar{3}m$ phases with decreasing channel diameter (Note that the difference in water channel diameter is not at scale in this schematic). The different shades of blue in the water channels indicate the lifetime of the intermolecular modes of water, with darker shades representing longer lifetimes. The orange region represents the lipid bilayer.

From an atomistic perspective, HBs in the system can be distinguished using a geometrical definition,^[^ ^52^ ^]^ allowing us to identify both water–water (w–w) and water–lipid (w–l) HBs. To investigate the influence of the water‐lipid interface, we analyze the average number of HBs per water molecule (*n_HB_*) as a function of the distance from the membrane midplane (**Figure** **4**a). Although the soft confinement imposed by monoolein reduces the entropic penalties typically associated with interfacial disruption of the HB network, steric hindrance imposed by the lipid matrix leads to a reduction in *n_HB_* by up to 14% at ≈1 nm from membrane center (Figure 4a). The region where we distinguish the undercoordination in the HB network is largely occupied by lipid head moieties as displayed by the density profile in (Figure S6, Supporting Information). Notably, the lipid headgroups are distributed according to a Gaussian profile centered around ≈1.5 nm from the membrane mid‐plane (see Figure S6, Supporting Information). In this region w–l HB increase monotonically as we approach the membrane center, up to distances of around 1 nm (Figure S7b, Supporting Information) where the number of w–l HBs is approximately 0.8 per molecule, only ≈50% lower than the w–w HB value of 1.5(Figure S7a, Supporting Information). Figure 4a shows that water molecules do not reach bulk‐like coordination levels until ≈2.4 nm (*x_c_*, in the Figure 4a). Taking 1.5 nm from the membrane center (center of the Gaussian of the lipid heads density profile Figure S6, Supporting Information) as the approximate position of the water‐lipid interface, our results indicate that water remains undercoordinated up to 0.9 nm beyond the interface, consistent with previous simulation studies on lipid systems.^[^ ^12^, ^39^ ^]^

When considering the effect of temperature, we distinguish a monotonic decrease of *n_HB_* at distances above ≈ 1.8 nm from the membrane center as temperature increases, while, results near the water‐lipid interface, i.e. at distances below this value, become noisy, with no clear pattern with temperature. Remarkably, the spatial extent of undercoordination remains unchanged across the investigated temperature range. In other words, bulk‐like hydrogen‐bonding behavior is consistently recovered only beyond ≈ 2.4 nm from the membrane midplane (*x_c_*, in the Figure 4a), independent of temperature. The temperature dependence of the HB network is more clearly revealed by the total number of HBs in the system, as shown in the inset of Figure 4a. This quantity decreases with increasing temperature and follows Arrhenius behavior, with an extracted activation energy of 1.48 kJ mol^−1^. We interpret this monotonic reduction as a fingerprint of the progressive weakening of the HB network with increasing temperature, as previously established.^[^ ^53^ ^]^ Specifically, the observed decrease in both τ_*streching*_ and τ_*libration*_ (Figure 3c,d) with temperature can be attributed to the reduction in the number of HBs per water molecule.

Molecular dynamics simulations reveal that the interfacial water fraction can be universally identified in these topologically distinct swollen mesophases at different temperatures. Practically, this enables the analytical estimation of the interfacial water fraction (*f*_*w*,*int*_) using a parameter‐free expression derived for swollen monolinolein‐SE systems^[^ ^12^ ^]^: $f_{w,int}=\frac{1}{\Gamma }\;\left(-\frac{0.543}{K_{0}}-2.092\right)$, where Γ  = *f_w_* *a* ^3^/(4π|χ|). Here *f_w_* denotes the total fraction of water in the system, *a* is the lattice parameter and χ is the Euler Poincaré characteristic, i.e., −8, −2, −4 for $Ia\bar{3}d,\;Pn\bar{3}m,$ and $Im\bar{3}m$ respectively.

This formulation allows for the quantitative disentanglement of interfacial water's contribution to the dynamics of intermolecular vibrational modes, across temperature and topological distinct regimes. The results for all systems and temperatures investigated are presented in Figure 4b,c. A general trend emerges: the lifetimes of the intermolecular vibrational modes increase with the interfacial water fraction. Interfacial water, through its strong HB with polar moieties of the lipid headgroup,^[^ ^7^ ^]^ disrupts the HB network of water (Figure 4a) and leads to an increase in the lifetime of the intermolecular bonds. Hence, systems with a larger interfacial water population exhibit longer lifetimes of the intermolecular vibrational modes. This contrasts with findings in granular matter,^[^ ^54^ ^]^ where a more regular tetrahedral network, i.e., higher *n_HB_*, correlates with dynamical slowing down. In soft nanoconfinement, however, the presence of hydrogen bonding between chemically distinct species introduces additional complexity, often giving rise to a counterintuitive behavior.

Interestingly, fitting the lifetimes versus the interfacial water fraction (*f*_*w*,*int*_) reveals a nonlinear relationship with the interfacial water fraction (Figure 4 b,c), which persists consistently across the investigated temperature range. Water motion is coupled and strongly correlated^[^ ^55^ ^]^ and, we hypothesize that this nonlinearity arises from the correlation within the confined system, where cooperative interactions and structural heterogeneity modulate the water dynamics in a non‐additive manner.

Overall, our results display a common picture of the dynamics of both intermolecular modes of water confined within the topologically distinct environment of IBCPs (Figure 4d). In particular, upon an increase in confinement, i.e., increase of *f*_*w*,*int*_, we find an increase in the lifetimes of both stretching and librational modes (Figure 4b–d). The mechanisms by which the cooperative HB network of water is disrupted when entering into contact with rough, inhomogeneous and curvature‐rich surfaces, such as the water‐lipid interface present in swollen mesophases, are complex. This complexity is corroborated by our results, which show a non‐linear dependence of these lifetimes on *f*_*w*,*int*_ maintained across all investigated temperatures. These findings underscore that soft nanoconfinement has a profound and nontrivial influence on the dynamics of the HB network.

## 3 Conclusion

Using SAXS, THz absorption spectroscopy, and atomistic MD simulations, we have investigated the temperature‐dependent behavior of phase transitions and intermolecular vibrational modes of water confined within swollen dimodan‐water IBCPs.

Building upon established studies of swollen mesophases at room temperature, our work offers a systematic exploration of how temperature modulates both structural and dynamical properties in these topologically distinct systems. In particular, it highlights the critical role of SE as a co‐surfactant in modulating lipid packing, as revealed by via SAXS. The findings demonstrate the stabilization of the $Pn\bar{3}m$ phase across a broad temperature range and uncover a nontrivial re‐entrant phase transition upon heating. We hypothesize that this phenomenon originates from different specific surfaces associated with the different mesophase symmetries.

The analysis of nanoconfined water ultrafast dynamics, experimentally carried out by THz absorption spectroscopy, underscores the distinct nature of nanoconfined water's hindered translations and rotations, stretching and libration modes. The temperature behavior of these modes exhibits no discernible topology dependence within the resolution of our measurements. The stretching mode consistently exhibits longer lifetimes than the librational mode. Interestingly, despite this slower relaxation, the activation energy extracted from the Arrhenius temperature dependence is twice as low for the stretching mode than for libration, indicating more temperature sensitivity.

To disentangle the role of confinement, atomistic simulations reveal that the spatial extent of the disruption of the structural features in the interfacial water HB network remain invariant across the temperature range studied. This finding enables an analytical quantification of the interfacial water fraction, which correlates with an increase in the lifetimes of both intermolecular vibrational modes. Notably, this relationship is nonlinear, indicating that the influence of interfacial water on vibrational dynamics is not simply additive or proportional. This nonlinearity points to a complex interplay between geometry, hydration level, and collective molecular behavior in soft nanoconfined environments.

These findings open the door to future investigations on the role of cooperativity in the structure and dynamics of the HB network of nanoconfined water. On this front, the recently introduced technique, correlated vibrational spectroscopy,^[^ ^56^ ^]^ enables the disentanglement of spectral features from interacting and non‐interacting water molecules, considering the influence of electronic charge transfer and nuclear quantum effects. This technique may provide a broader picture of the variation on the HB network in samples with different interfacial water populations across increasing temperatures and its use will be attempted in our future efforts.

Taken together, our results advance our understanding of hydrogen‐bond network dynamics in swollen mesophases, shedding light on the molecular factors that govern their stability and guide the design of optimized systems for biomedical, pharmaceutical, and food applications.

## 4 Experimental Section

### Sample Preparation

Swollen mesophases were obtained by equilibrating a mixture of Dimodan U/J, sugar ester, and water. Dimodan U/J (a gift from Danisco, Denmark) is a food additive, commercially available as monolinolein monoglyceride with a purity of more than 98 wt.%. No purification steps were implemented before use. Sugar ester S‐1670 is a food additive and was obtained from Mitsubishi‐Kagaku Foods Corporation (Japan). The specific batch used for this study has the lot number 3 × 178Y14 and consists of 75% monoester and 25% di‐, tri‐, and polyesters. In the following it will be referred to as SE (sugar ester). Ultrapure water was used in the preparation of all the lipid mesophases. The initial step of the preparation involved the homogenization of Dimodan U/J with SE. Dimodan U/J was melted at 60 °C, and the corresponding mass of SE was slowly added while vigorously mixing with a magnetic stirrer. The mixture was then kept in an oil bath at 60 °C under constant and gentle magnetic stirring until the SE was fully dissolved. The mesophases were prepared using two gas‐tight syringes, one containing water and the other the mixture of Dimodan U/J and SE. The two components were mixed with the syringes until homogenized. After preparation, the samples were left to equilibrate overnight at room temperature. For SAXS measurements, the samples were loaded into 1.5 mm diameter quartz capillaries and sealed with epoxy glue. For THz studies the samples were stored in sealed vials.

### Small Angle X‐Rays Diffraction (SAXS)

SAXS measurements were conducted using a Rigaku microfocused X‐ray SAXS instrument, equipped with a Ni‐filtered Cu Kα radiation source with a wavelength (λ) of 1.54 Å, operating at 45 kV and 88 mA. The diffracted X‐ray signal was collected using a gas‐filled 2D detector. The scattering vector q = (4π/λ) sinθ, with 2θ as the scattering angle, was calibrated using silver behenate. The presented data were collected in the range of 0.05 < q < 0.45 Å^−1^, representing the azimuthally averaged results. For every temperature acquisition, the samples were equilibrated for 30 minutes prior to measurement, and the scattered light was collected for 45 consecutive minutes.

The Bragg reflections of the IBCPs correspond to space groups *Q* ^230^ ($Ia\bar{3}d$), *Q* ^224^ ($Pn\bar{3}m$) and *Q* ^229^ ($Im\bar{3}m$) and follow spacings determined by the relation: $d_{h,k,l}=\frac{a}{\sqrt{h^{2}+k^{2}+l^{2}}}\;,$ where *h*, *k*, *l* denote the Miller indeces of the refractive planes, and *a* is the lattice parameter.

The average channel diameter of the IBCPs is calculated following the relation^[^ ^57^ ^]^:

(3) $$d=\sqrt{\frac{A_{0}}{2\pi \left|\chi \right|}}\;a-l$$

where *a* is the lattice parameter, *A*_0_ = 3.091, 1.919, 2.345, is the normalized unit cell surface area, and χ  = −8, −2, −4, is the Euler Poincaré characteristic, the values of both parameters are for $Ia\bar{3}d,\;Pn\bar{3}m$ and $Im\bar{3}m$ respectively. *l* is the lipid length which is calculated,^[^ ^58^ ^]^

(4) $$\phi_{L}=2A_{0}\left(\frac{l}{a}\right)+\frac{4\pi \chi }{3}{\left(\frac{l}{a}\right)}^{3}$$

where ϕ _*L*_ is the lipid volume fraction.

### THz FTIR Measurements

THz/far infrared (THz/FIR) measurements of lipid mesophases were carried out at the infrared beamline SISSI Bio at the Elettra synchrotron facility in Trieste, Italy.^[^ ^59^ ^]^ A Fourier transform infrared spectrometer (FTIR, Vertex 70 V, Bruker) equipped with an external He‐cooled bolometer (Infrared Laboratories, Inc., Tucson, USA) for light detection was employed. The sample was positioned on the temperature‐controlled single‐reflection Diamond ATR crystal (Platinum ATR, Bruker GmbH) covered with a vacuum‐tight lid. Spectra were acquired in the range of 50–550 cm^−1^ with a resolution of 2 cm^−1^. Each data point represents the average of 5 spectra, with each spectrum obtained as the average of 128 scans at 40 kHz at the set temperature. Prior to the measurement, the sample was allowed to equilibrate for 5 minutes on the sample holder.

The temperature dependent absorption coefficient (α(ν, *T*)) of the sample is calculated with the Beer‐Lambert law^[^ ^60^ ^]^:

(5) $$\alpha \;\left(\nu ,T\right)=\frac{1}{d_{s}}log\left(\frac{I_{0}\left(\nu ,T\right)}{I\left(\nu ,T\right)}\right)$$

where *d_s_* is the thickness of the sample, *I*_0_ and *I* are the incident and transmitted intensities and ν is the frequency of the incident light, between 50 and 550 cm^−1^. The temperature‐dependent sample thickness is calculated considering the refractive index as previously established:^[^ ^61^ ^]^

(6) $$d_{s}=\frac{\lambda }{2\pi \sqrt{n_{C}^{2}\cdot \sin{\left(\theta \right)}^{2}-n_{s}^{2}}}$$

where $\theta =\frac{\pi }{4}$ is the angular orientation of the incident beam, *n_C_* and *n_s_* are the refractive indexes of the diamond crystal in the ATR unit and the sample respectively. As a practical approximation, we use their values at 298 K and at visible wavelengths. For the sake of simplicity, based on values previously reported,^[^ ^62^ ^]^ we fixed *n_C_* = 2.42 and *n_s_* =  1.47, 1.44, 1.41, for 30%,40%, and 50% of water content respectively.^[^ ^12^ ^]^ For the purpose of the analysis, the influence of SE content in neglected, as the reported variation in refractive index is less than 1% for a 15% change in SE concentration.

For all samples we subtracted the absorption spectrum of the Dimodan SE mixture (α_*m*_(ν,*T*)) weighed by its volume fraction (ϕ _*m*_), to isolate the contribution of water to the overall spectrum:

(7) $$\Delta \alpha \;\left(\nu ,T\right)=\alpha \left(\nu ,T\right)-\phi_{m}\alpha_{m}\left(\nu ,T\right)$$

The modelling was performed as indicated in the main text. Specifically, the low frequency mode (α_*LF*_) was described with the following formula:

(8) $$\alpha_{LF}\left(\nu \right)=\frac{a_{0}e^{-\frac{\nu }{\nu_{0}}}}{\pi \left(\nu^{2}+\frac{\omega_{0}^{2}}{\pi^{2}}\right)}\;\nu^{2}$$

where *a*_0_, ν_0_, ω_0_ are the amplitude, center frequency, and width of the mode. The overlapping high frequency mode was modelled with the sum of two contributions:^[^ ^12^, ^43^ ^]^

(9) $$\alpha_{HF}\;\left(\nu \right)=t_{2}+t_{3}$$

where

(10) $$t_{i}=\frac{a_{i}\omega_{i}^{2}}{4\pi^{3}\left({\left(\nu b_{i}^{2}+\frac{\omega_{i}^{2}}{4\pi }-\nu^{2}\right)}^{2}+\frac{\omega_{i}^{2}}{\pi^{2}}\nu^{2}\right)}\;\nu^{2}$$

The parameters used in α_*HF*_ and α_*LF*_, are presented in.^[^ ^12^ ^]^

### Atomistic Molecular Dynamics Simulation

Molecular dynamics simulations were performed with GROMACS 2022.4.^[^ ^63^ ^]^ The unit cell was obtained from equilibrated lamellar monolinolein systems at 54% hydration and 298 K, as reported in a previous study.^[^ ^12^ ^]^ From this starting configuration we simulated the system at increasing temperatures ranging from 308 to 338 K in steps of 5 K. Langevin integrator was used to maintain the temperature with a damping constant of 0.5 ps^−1^. Lennard Jones interactions and short‐range electrostatics were treated with a cut‐off at 1.2 nm, whereas the long‐range electrostatics were implemented with particle mesh Ewald summation. The time step was set to 2 fs, which was enabled due to the constraint of the covalently bonded hydrogen atoms via LINCS algorithm. The equilibration of the system at the different temperatures was achieved with a 2 ns pressure constant simulation, at 1 bar via C‐rescale barostat.^[^ ^64^ ^]^ Followed by a 2 ns canonical ensemble simulation enabling the further equilibration of the temperature in the system (the equilibration of the box size and the temperature is shown in Figures S8,S9, Supporting Information). The OPC model was used for water while the lipids were simulated with a combination of gromos 54A7 force field and in house parametrization for the lipid heads, for a good characterization of hydroxyl lipid groups water interactions.^[^ ^12^ ^]^

The analysis of the hydrogen bonds was performed with 10 ps long NVT simulations, sampling in every step (2 fs). We then calculated the HB with both lipids and water considering the established geometric criteria for the HB definition.^[^ ^52^ ^]^ Bulk values of the average number of HB per water molecule were calculated from equilibrated 5 nm boxes of water modelled using the OPC model.

## Conflict of Interest

The authors declare no conflict of interest.

## Author Contributions

E.Z.B. and S.R.A. contributed equally to this work. E.Z.B. performed conceptualization, methodology, data curation, wrote original draft, wrote, reviewed, and edited the manuscript, and performed visualization; S.R.A. performed conceptualization, investigation; L.B. and L.V performed investigation; P.Z., F.P., and H.V. performed investigation, wrote, reviewed, and edited the draft; R.M. performed conceptualization, resources, wrote, reviewed, and edited the manuscript, performed supervision, and acquired funding acquisition.

## Supporting information



Supporting Information

## Data Availability

The data that support the findings of this study are available from the corresponding author upon reasonable request.
