# Supplementary material for: Ultrafast Intermolecular Dynamics of Nanoconfined Water in Swollen Lipid Cubic Mesophases
Source: Small. 2025 Oct 3;21(46):e08744. doi: 10.1002/smll.202508744 (PMC12632434; doi:10.1002/smll.202508744)
Supplement: Supplementary file 1 — Supporting Information [file SMLL-21-e08744-s001.docx]

Supporting Information for:

**Ultrafast Intermolecular Dynamics of Nanoconfined Water in Swollen Lipid Cubic Mesophases**

Eva Zunzunegui-Bru^1,+^, Serena Rosa Alfarano^1,+^, Patrick Züblin^1^, Laura Baraldi^1^, Hendrik Vondracek^2,4^, Federica Piccirilli^2,3^, Lisa Vaccari^2^, and Raffaele Mezzenga^1,5,*^

^1^Department of Health Sciences and Technology, ETH Zurich, Zurich, 8092 Switzerland

^2^Elettra Sincrotrone Trieste, Strada Statale 14 km 163.5 in Area Science Park Basovizza, Trieste, 34149 Italy

^3^ AREA Science Park, Padriciano, 99 34149 Trieste – Italy

^4^Diamond Light Source. MIRIAM Infrared Beamline B22. Harwell Science and Innovation Campus. Didcot. United Kingdom. OX11 0DE

^5^Department of Materials, ETH Zurich, Zurich, 8092 Switzerland

* corresponding author E-mail: raffaele.mezzenga@hest.ethz.ch

+ E.Z.B. and S.R.A. contributed equally to this work.

**
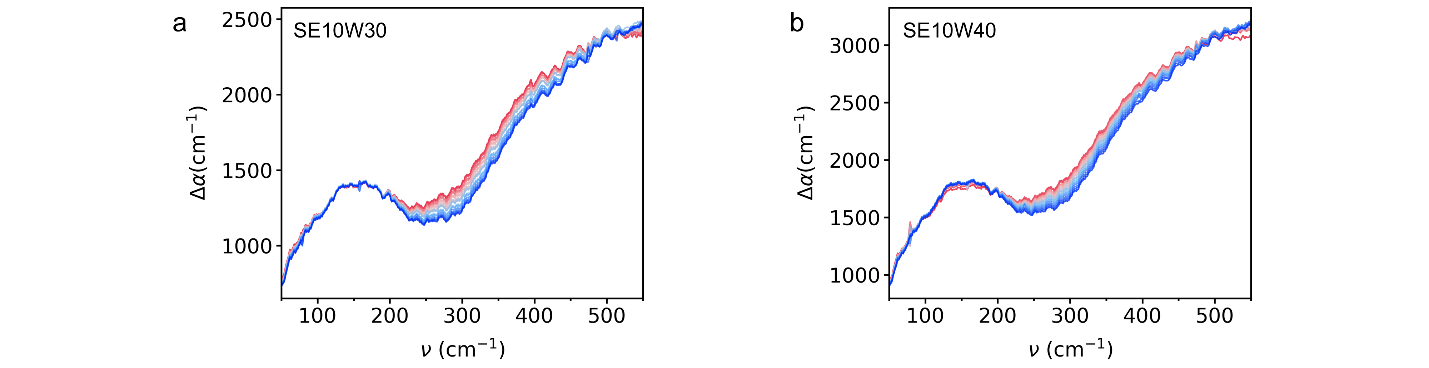
**

**Figure S1.** Water THz absorption spectra ($\Delta\alpha$) for **a)** SE10-W30, **b)** SE10-W40 sample across the temperature range 298-340 K.

**Table S1.** Fitting parameters of the THz spectra for **SE10-W30**. All parameters are given in (cm⁻¹). The vibrational modes of water are referred to as 'stret' (stretching), 'lib' (libration), and 'LF' (Debye low-frequency mode), 'HF' (overlapping high frequency modes).

| **T(K)** | $\boldsymbol{\nu}_{\boldsymbol{0,stret}}$ | $\boldsymbol{a}_{\boldsymbol{0,stret}}$ | $\boldsymbol{\omega}_{\boldsymbol{0,stret}}$ | $\boldsymbol{\nu}_{\boldsymbol{0,lib}}$ | $\boldsymbol{a}_{\boldsymbol{0,lib}}$ | $\boldsymbol{\omega}_{\boldsymbol{0,lib}}$ | $\boldsymbol{a}_{\boldsymbol{LF}}$ | $\boldsymbol{a}_{\boldsymbol{HF}}$ |
| --- | --- | --- | --- | --- | --- | --- | --- | --- |
| 298 | 144.20 ± 0.36 | 8609.70 ± 88.49 | 432.31 ± 6.21 | 419.28 ± 1.15 | 4923.95 ± 90.76 | 546.61 ± 15.36 | 192.30 ± 2.16 | 39.55 ± 0.25 |
| 301 | 144.19 ± 0.37 | 8558.15 ± 90.30 | 433.70 ± 6.32 | 419.17 ± 1.34 | 5040.35 ± 99.46 | 553.92 ± 16.73 | 194.11 ± 2.18 | 39.38 ± 0.27 |
| 304 | 144.19 ± 0.36 | 8484.42 ± 88.69 | 436.54 ± 6.33 | 417.76 ± 1.10 | 5119.03 ± 91.14 | 553.81 ± 14.75 | 195.11 ± 2.21 | 39.35 ± 0.25 |
| 307 | 144.20 ± 0.36 | 8489.53 ± 91.50 | 442.83 ± 6.33 | 418.24 ± 1.09 | 5422.69 ± 96.48 | 579.63 ± 14.99 | 195.10 ± 2.16 | 38.88 ± 0.24 |
| 310 | 144.20 ± 0.36 | 8394.65 ± 85.25 | 446.67 ± 6.28 | 417.17 ± 1.03 | 5530.52 ± 86.10 | 585.36 ± 13.90 | 196.64 ± 2.08 | 38.80 ± 0.22 |
| 313 | 144.20 ± 0.36 | 8343.65 ± 84.63 | 453.21 ± 6.40 | 416.71 ± 1.06 | 5744.32 ± 90.88 | 600.86 ± 14.29 | 197.04 ± 2.07 | 38.48 ± 0.23 |
| 316 | 144.20 ± 0.36 | 8388.81 ± 84.55 | 465.45 ± 6.35 | 416.28 ± 1.13 | 6033.56 ± 101.34 | 622.30 ± 14.60 | 196.46 ± 2.08 | 38.54 ± 0.26 |
| 319 | 144.20 ± 0.36 | 8276.13 ± 88.28 | 466.21 ± 6.43 | 416.57 ± 0.99 | 6258.38 ± 89.63 | 637.33 ± 13.15 | 197.46 ± 2.08 | 37.69 ± 0.26 |
| 322 | 144.20 ± 0.37 | 8199.33 ± 84.53 | 474.27 ± 6.75 | 416.79 ± 0.98 | 6577.82 ± 92.12 | 658.72 ± 13.07 | 198.12 ± 2.06 | 37.05 ± 0.24 |
| 325 | 144.20 ± 0.37 | 8153.00 ± 83.68 | 480.37 ± 6.65 | 416.30 ± 1.04 | 6753.50 ± 103.57 | 671.96 ± 13.34 | 198.57 ± 2.05 | 36.93 ± 0.26 |
| 328 | 144.20 ± 0.38 | 8070.75 ± 85.24 | 486.07 ± 7.17 | 416.77 ± 1.29 | 7074.06 ± 132.32 | 695.20 ± 16.07 | 199.06 ± 2.08 | 36.15 ± 0.32 |
| 331 | 144.20 ± 0.37 | 8038.42 ± 83.53 | 492.49 ± 6.90 | 417.16 ± 0.99 | 7394.21 ± 99.64 | 719.25 ± 12.92 | 199.16 ± 2.06 | 35.62 ± 0.26 |
| 334 | 144.20 ± 0.38 | 7933.36 ± 82.89 | 495.68 ± 7.13 | 417.41 ± 1.18 | 7667.79 ± 125.68 | 737.04 ± 14.23 | 200.37 ± 2.07 | 34.95 ± 0.33 |
| 337 | 144.20 ± 0.39 | 7887.82 ± 85.41 | 506.24 ± 7.41 | 417.01 ± 1.14 | 7922.84 ± 122.52 | 752.03 ± 14.09 | 200.28 ± 2.10 | 34.54 ± 0.31 |
| 340 | 144.20 ± 0.40 | 7796.99 ± 84.74 | 510.22 ± 7.54 | 418.06 ± 1.03 | 8327.60 ± 112.37 | 777.76 ± 13.05 | 200.72 ± 2.08 | 33.54 ± 0.29 |

**
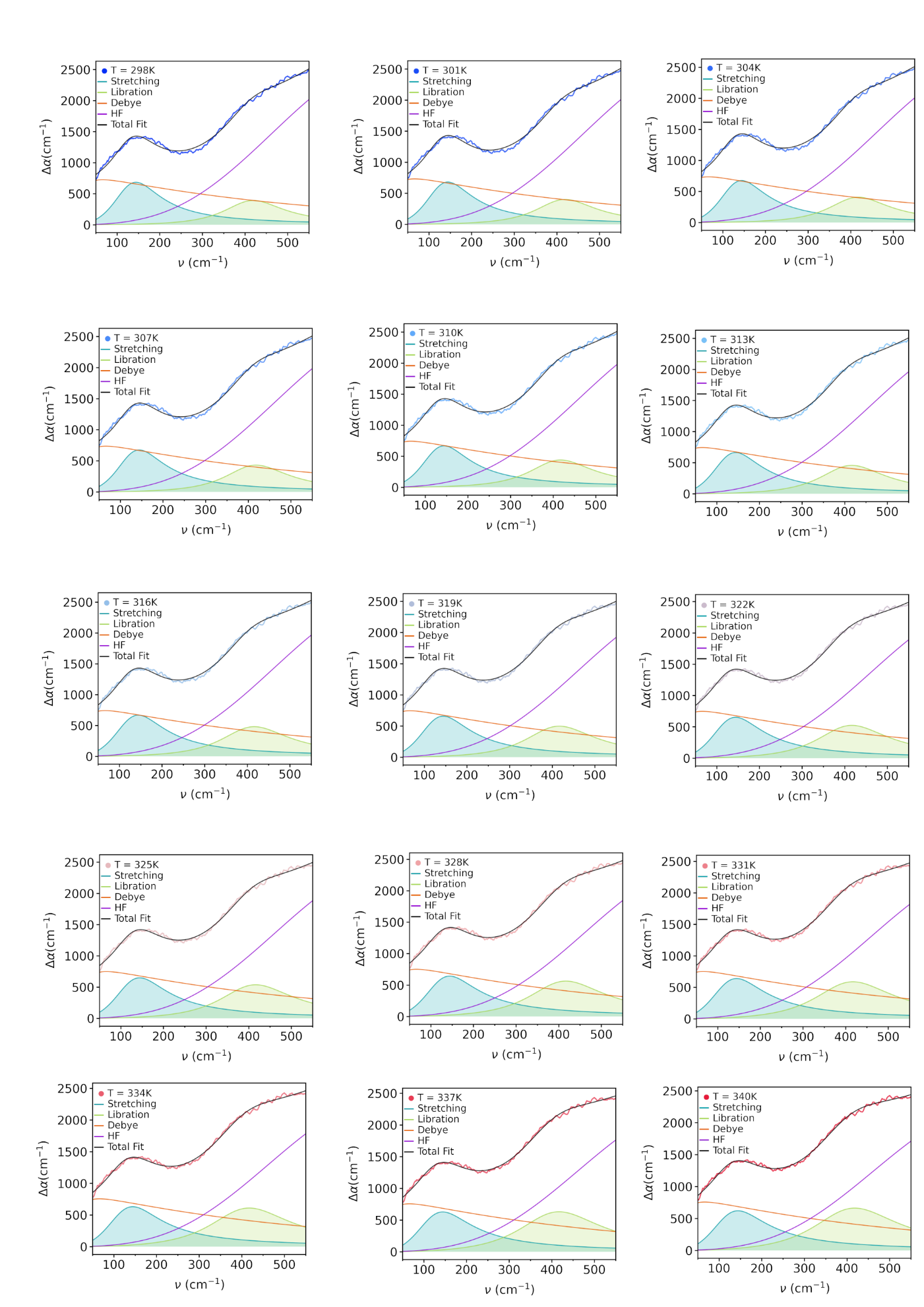
Figure S2.** Fit for the THz absorption spectra of water for **SE10-W30** across temperature range 298-340 K.

**Table S2.** Fitting parameters of the THz spectra for **SE10-W40**. All parameters are given in (cm⁻¹). The vibrational modes of water are referred to as 'stret' (stretching), 'lib' (libration), and 'LF' (Debye low-frequency mode), 'HF' (overlapping high frequency modes).

| **T(K)** | $\boldsymbol{\nu}_{\boldsymbol{0,stret}}$ | $\boldsymbol{a}_{\boldsymbol{0,stret}}$ | $\boldsymbol{\omega}_{\boldsymbol{0,stret}}$ | $\boldsymbol{\nu}_{\boldsymbol{0,lib}}$ | $\boldsymbol{a}_{\boldsymbol{0,lib}}$ | $\boldsymbol{\omega}_{\boldsymbol{0,lib}}$ | $\boldsymbol{a}_{\boldsymbol{LF}}$ | $\boldsymbol{a}_{\boldsymbol{HF}}$ |
| --- | --- | --- | --- | --- | --- | --- | --- | --- |
| 298 | 145.20 ± 0.30 | 11583.74 ± 98.28 | 481.82 ± 5.35 | 415.38 ± 1.02 | 7049.95 ± 98.13 | 617.32 ± 12.81 | 228.82 ± 2.39 | 50.27 ± 0.28 |
| 301 | 145.20 ± 0.30 | 11616.32 ± 100.09 | 484.32 ± 5.42 | 415.84 ± 1.06 | 7358.31 ± 109.11 | 634.45 ± 13.27 | 229.44 ± 2.43 | 49.85 ± 0.28 |
| 304 | 145.20 ± 0.30 | 11609.33 ± 96.95 | 489.47 ± 5.52 | 415.18 ± 0.97 | 7509.74 ± 116.96 | 643.62 ± 13.50 | 229.96 ± 2.37 | 49.92 ± 0.29 |
| 307 | 145.19 ± 0.31 | 11582.88 ± 100.45 | 497.68 ± 5.64 | 414.77 ± 1.04 | 7745.07 ± 112.69 | 655.99 ± 13.50 | 229.96 ± 2.50 | 49.73 ± 0.29 |
| 310 | 145.20 ± 0.30 | 11518.91 ± 95.22 | 501.63 ± 5.53 | 414.90 ± 0.92 | 8055.36 ± 101.71 | 676.09 ± 12.19 | 230.51 ± 2.33 | 49.09 ± 0.27 |
| 313 | 145.20 ± 0.30 | 11467.72 ± 95.79 | 507.77 ± 5.66 | 414.79 ± 0.99 | 8323.93 ± 110.58 | 694.13 ± 12.91 | 230.41 ± 2.35 | 48.66 ± 0.28 |
| 316 | 145.20 ± 0.31 | 11374.95 ± 107.73 | 512.79 ± 5.92 | 415.66 ± 1.04 | 8641.77 ± 111.50 | 715.44 ± 12.76 | 230.98 ± 2.54 | 47.97 ± 0.30 |
| 319 | 145.20 ± 0.30 | 11330.22 ± 93.70 | 517.55 ± 5.72 | 414.73 ± 0.92 | 8829.88 ± 112.86 | 725.63 ± 12.42 | 230.75 ± 2.29 | 47.69 ± 0.28 |
| 322 | 145.20 ± 0.31 | 11298.28 ± 95.93 | 525.92 ± 5.84 | 415.01 ± 0.93 | 9133.22 ± 111.10 | 744.44 ± 12.07 | 229.52 ± 2.43 | 47.21 ± 0.28 |
| 325 | 145.20 ± 0.31 | 11222.27 ± 95.13 | 531.14 ± 6.05 | 415.05 ± 0.90 | 9419.48 ± 111.16 | 760.51 ± 11.90 | 229.25 ± 2.34 | 46.56 ± 0.29 |
| 328 | 145.20 ± 0.32 | 11132.36 ± 98.23 | 534.35 ± 6.29 | 415.76 ± 0.98 | 9775.89 ± 129.49 | 786.10 ± 13.09 | 229.73 ± 2.36 | 45.85 ± 0.32 |
| 331 | 145.20 ± 0.32 | 11025.28 ± 96.05 | 542.44 ± 6.35 | 415.79 ± 0.98 | 10015.27 ± 120.76 | 798.84 ± 12.21 | 230.03 ± 2.35 | 45.28 ± 0.31 |
| 334 | 145.20 ± 0.33 | 10950.47 ± 99.48 | 548.57 ± 6.88 | 415.58 ± 1.14 | 10322.87 ± 155.82 | 817.61 ± 14.36 | 230.30 ± 2.39 | 44.79 ± 0.38 |
| 337 | 145.20 ± 0.33 | 10794.47 ± 94.53 | 553.17 ± 6.58 | 416.42 ± 0.95 | 10654.76 ± 127.23 | 840.94 ± 12.33 | 230.82 ± 2.29 | 43.81 ± 0.34 |
| 340 | 145.20 ± 0.36 | 10371.74 ± 99.54 | 557.85 ± 7.23 | 418.08 ± 1.06 | 10966.20 ± 145.75 | 867.54 ± 13.97 | 233.24 ± 2.35 | 42.12 ± 0.36 |


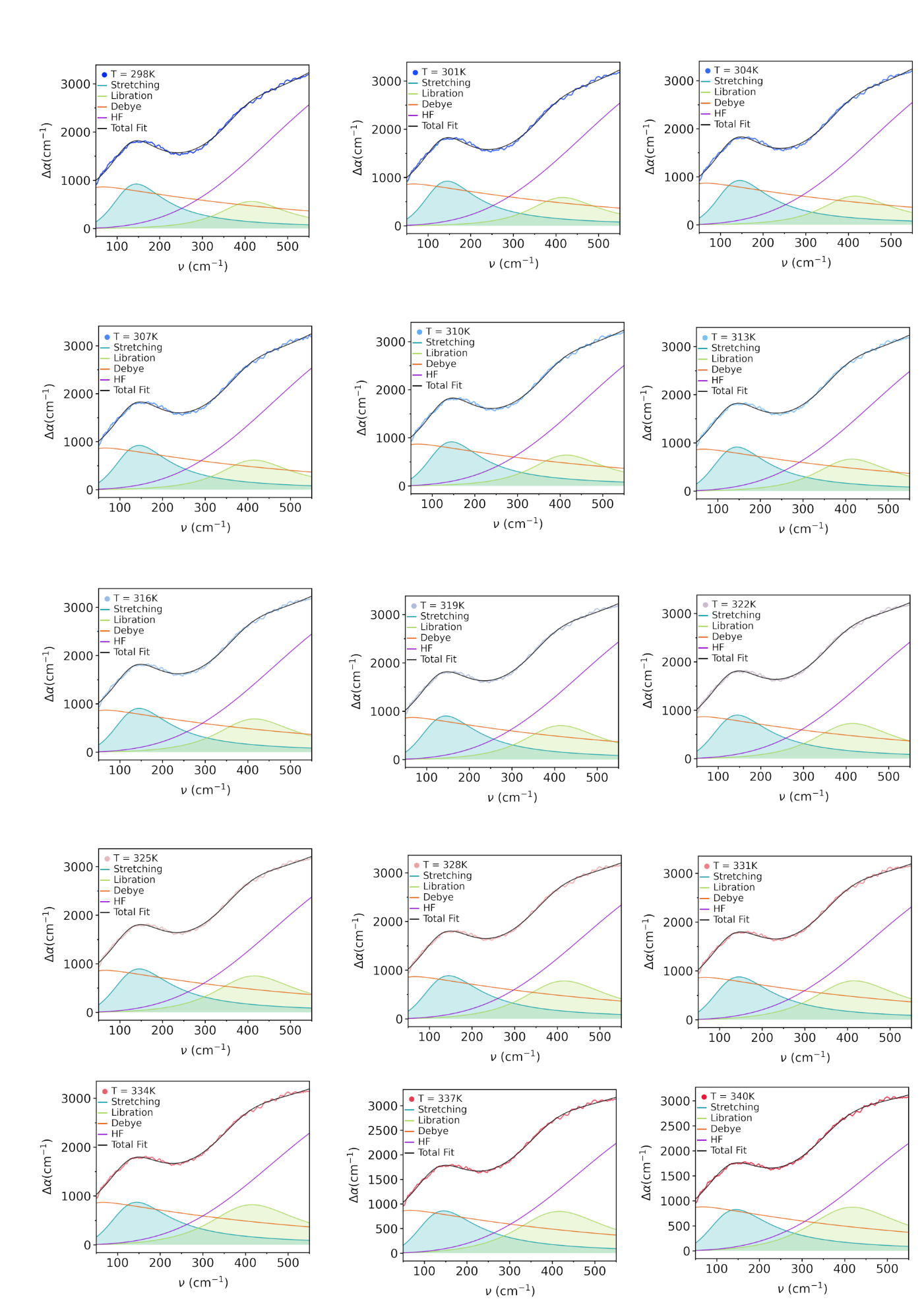
 **Figure S3.** Fit for the THz absorption spectra of water for **SE10-W40** across temperature range 298-340 K.

**Table S3.** Fitting parameters of the THz spectra for **SE20-W50**. All parameters are given in (cm⁻¹). The vibrational modes of water are referred to as 'stret' (stretching), 'lib' (libration), and 'LF' (Debye low-frequency mode), 'HF' (overlapping high frequency modes).

| **T(K)** | $\boldsymbol{\nu}_{\boldsymbol{0,stret}}$ | $\boldsymbol{a}_{\boldsymbol{0,stret}}$ | $\boldsymbol{\omega}_{\boldsymbol{0,stret}}$ | $\boldsymbol{\nu}_{\boldsymbol{0,lib}}$ | $\boldsymbol{a}_{\boldsymbol{0,lib}}$ | $\boldsymbol{\omega}_{\boldsymbol{0,lib}}$ | $\boldsymbol{a}_{\boldsymbol{LF}}$ | $\boldsymbol{a}_{\boldsymbol{HF}}$ |
| --- | --- | --- | --- | --- | --- | --- | --- | --- |
| 298 | 147.19 ± 0.19 | 16977.37 ± 92.24 | 569.75 ± 4.21 | 409.97 ± 0.55 | 12448.30 ± 87.50 | 747.94 ± 7.79 | 282.46 ± 2.30 | 63.87 ± 0.23 |
| 301 | 147.20 ± 0.19 | 17039.67 ± 83.61 | 570.53 ± 3.73 | 409.67 ± 0.51 | 12577.28 ± 86.74 | 752.44 ± 7.30 | 282.90 ± 2.06 | 63.94 ± 0.22 |
| 304 | 147.20 ± 0.19 | 17028.58 ± 90.95 | 573.69 ± 3.94 | 409.43 ± 0.53 | 12794.27 ± 101.95 | 764.07 ± 7.71 | 283.01 ± 2.18 | 63.61 ± 0.24 |
| 307 | 147.19 ± 0.19 | 17087.26 ± 84.26 | 578.51 ± 3.75 | 409.28 ± 0.54 | 13178.89 ± 89.84 | 777.48 ± 7.55 | 283.56 ± 2.05 | 63.00 ± 0.22 |
| 310 | 147.18 ± 0.19 | 17103.40 ± 84.05 | 581.43 ± 3.81 | 409.26 ± 0.53 | 13620.90 ± 96.13 | 795.35 ± 7.47 | 285.94 ± 2.02 | 62.17 ± 0.24 |
| 313 | 147.18 ± 0.19 | 17133.84 ± 83.39 | 588.21 ± 3.90 | 408.58 ± 0.54 | 13910.91 ± 94.83 | 804.73 ± 7.69 | 286.40 ± 2.00 | 61.84 ± 0.24 |
| 316 | 147.19 ± 0.19 | 17054.17 ± 96.27 | 593.10 ± 4.60 | 409.16 ± 0.71 | 14269.31 ± 121.57 | 822.50 ± 8.94 | 286.60 ± 2.24 | 60.84 ± 0.35 |
| 319 | 147.18 ± 0.21 | 17053.67 ± 97.45 | 599.73 ± 4.75 | 409.49 ± 0.67 | 14569.16 ± 116.60 | 838.88 ± 10.23 | 286.40 ± 2.22 | 60.23 ± 0.33 |
| 322 | 147.18 ± 0.21 | 16926.29 ± 94.79 | 605.76 ± 6.09 | 409.73 ± 0.64 | 14905.96 ± 148.40 | 855.14 ± 10.11 | 288.07 ± 2.25 | 59.46 ± 0.32 |
| 325 | 147.19 ± 0.20 | 16876.81 ± 95.41 | 611.26 ± 5.14 | 409.81 ± 0.55 | 15222.97 ± 108.01 | 872.13 ± 8.05 | 286.17 ± 2.30 | 58.54 ± 0.27 |
| 328 | 147.19 ± 0.20 | 16771.99 ± 90.15 | 616.37 ± 4.22 | 409.36 ± 0.52 | 15365.67 ± 102.89 | 883.60 ± 7.36 | 286.06 ± 2.13 | 58.20 ± 0.24 |
| 331 | 147.19 ± 0.20 | 16648.53 ± 101.07 | 618.54 ± 4.76 | 410.77 ± 0.57 | 15882.32 ± 116.52 | 912.79 ± 7.92 | 285.34 ± 2.55 | 56.61 ± 0.27 |
| 334 | 147.20 ± 0.20 | 16444.19 ± 86.75 | 623.29 ± 4.69 | 410.84 ± 0.57 | 16089.77 ± 114.51 | 927.83 ± 8.15 | 286.14 ± 2.04 | 55.84 ± 0.27 |
| 337 | 147.20 ± 0.21 | 16262.75 ± 110.77 | 625.87 ± 5.04 | 411.54 ± 0.56 | 16434.29 ± 110.96 | 951.68 ± 8.00 | 286.14 ± 2.45 | 54.71 ± 0.27 |
| 340 | 147.20 ± 0.21 | 16048.30 ± 84.46 | 628.00 ± 4.44 | 412.20 ± 0.69 | 16774.27 ± 127.56 | 973.03 ± 9.23 | 286.23 ± 1.89 | 53.42 ± 0.31 |


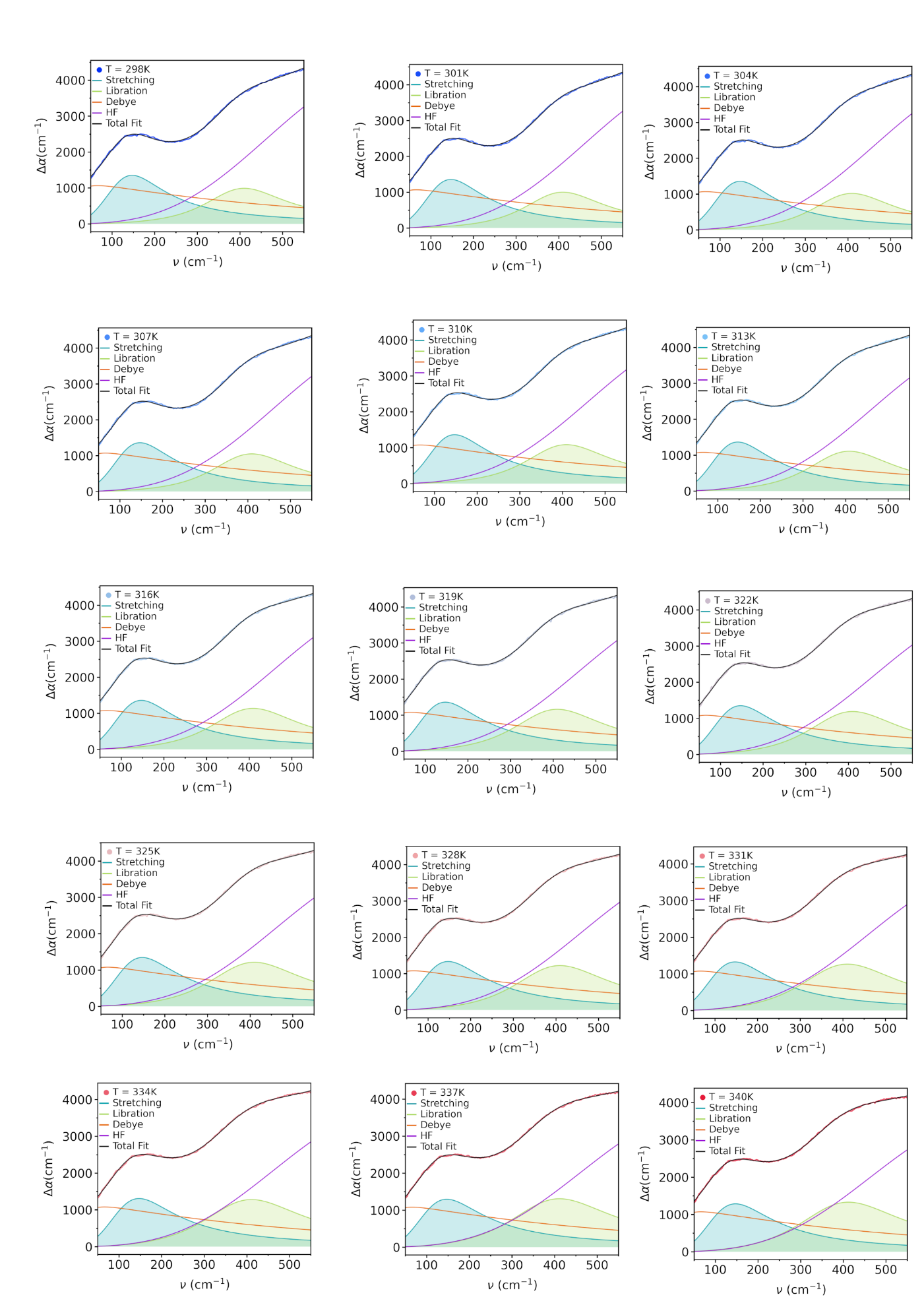
**Figure S4.** Fit for the THz absorption spectra of water for **SE20-W50** across temperature range 298-340 K.


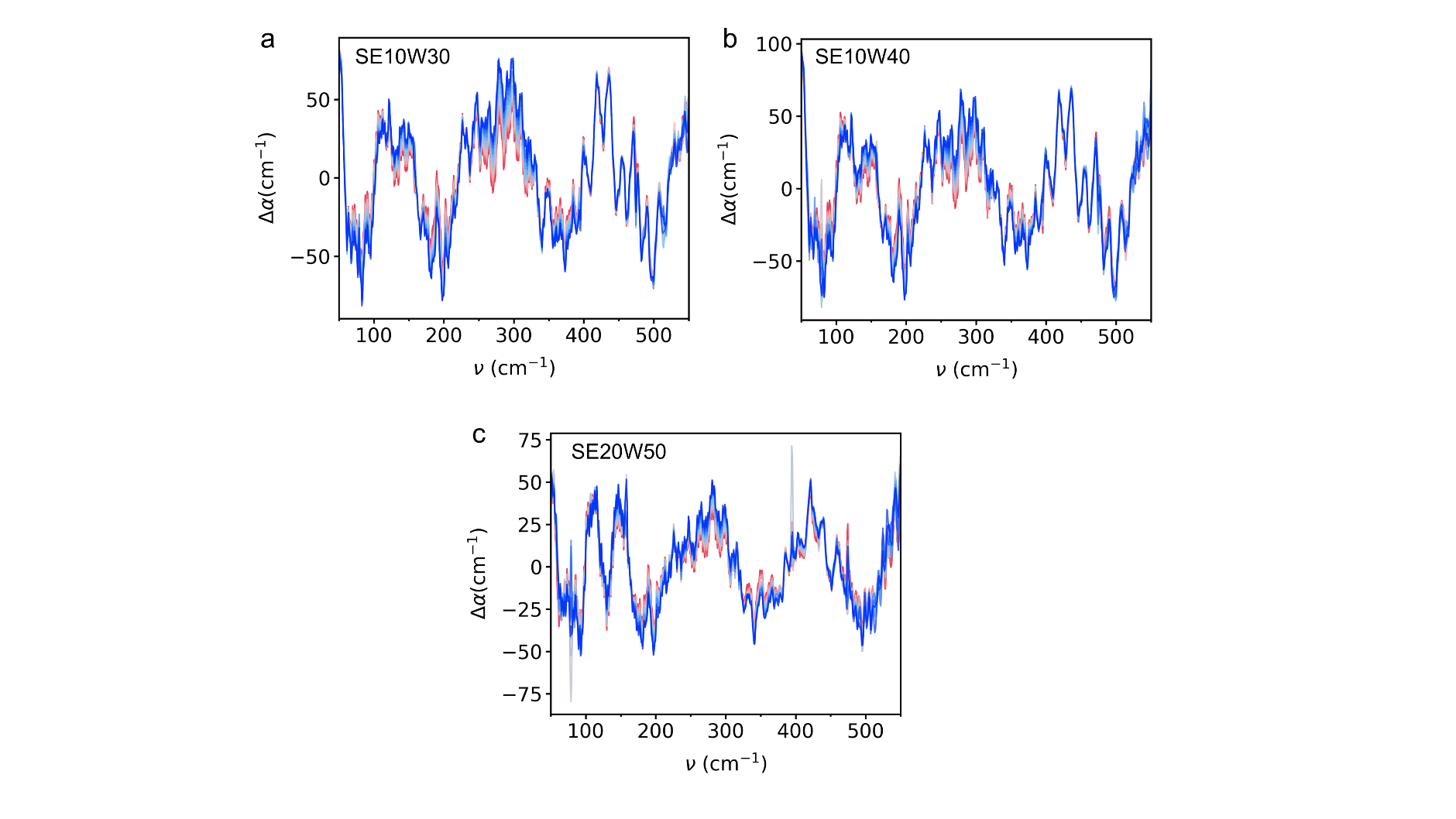


**Figure S5.** Residuals from the fit for all investigated samples, **a)** SE10W30, **b)** SE10W40 and **c)** SE20W50. The maximum residual value is approximately 50 cm⁻¹, corresponding to a relative error of $\sim$1.7%.


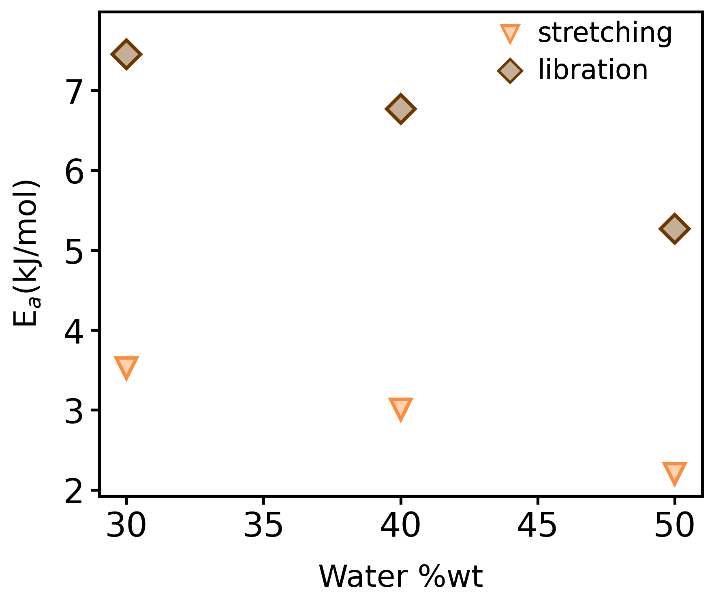


**Figure S6.** Activation energies obtained from the Arrhenius temperature dependence of the lifetimes of the stretching and libration modes of water. The results indicate a decrease in the energy with increasing water content for both modes.


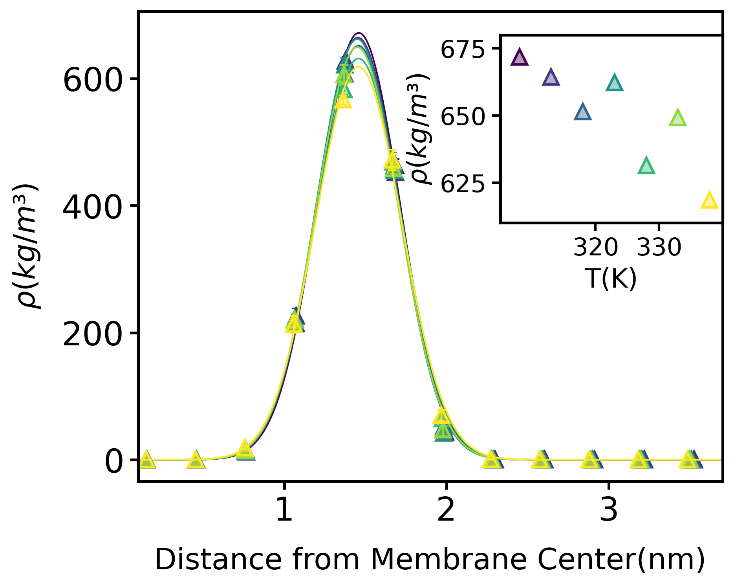


**Figure S7.** Density profile ($\rho$) of the lipid headgroups as a function of distance from the membrane center. The color code follows that used in the main text for the temperature range 308–338 K. *Inset:* Position of the center of the Gaussian fit to the headgroup density as a function of temperature. A decrease in peak density with increasing temperature is observed, consistent with enhanced thermal fluctuations and greater delocalization of the headgroups within the bilayer.


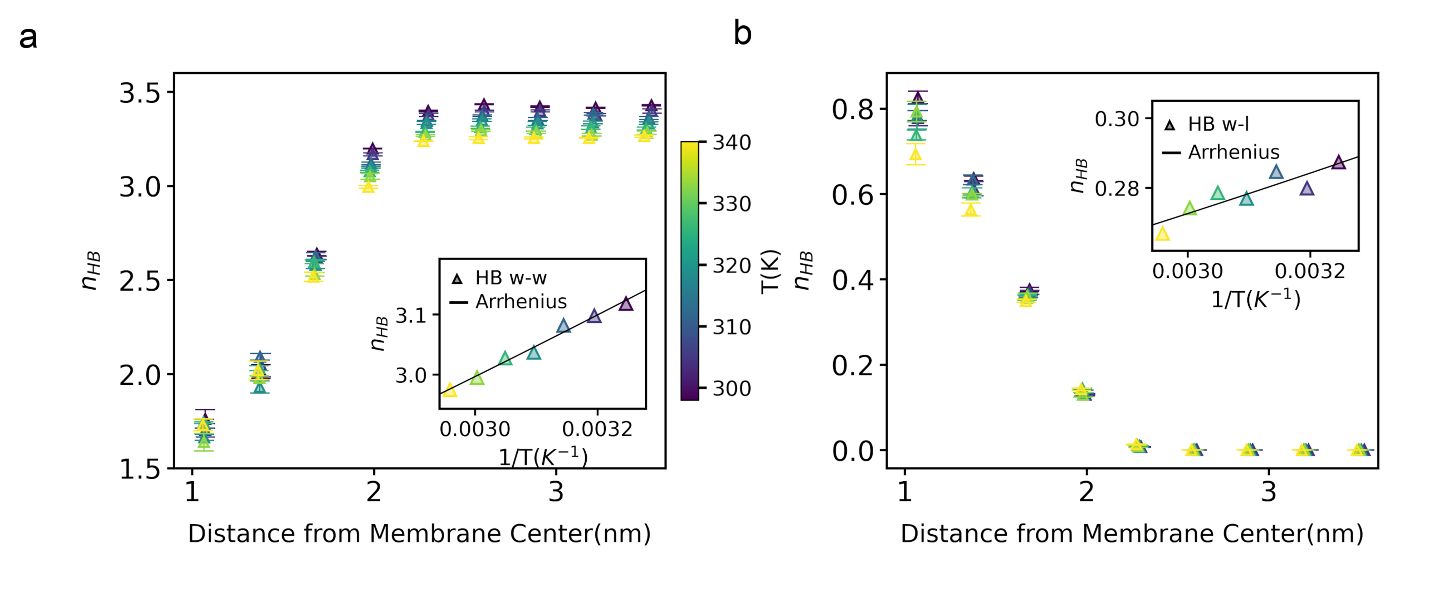


**Figure S8. a)** Average number of water-water (w-w) hydrogen bonds per water molecule as a function of the distance from the membrane center. The color code follows that used in the main text for the temperature range 308–338 K. A plateau is reached at approximately 2.4 nm, consistent with the results shown in Figure 4a of the main text. Inset: average number of w-w HB as a function of temperature. The black line represents an Arrhenius fit, yielding an activation energy of 1.26 $\pm$ 0.02 kJ/mol. **b)** Average number of water-lipid (w-l) hydrogen bonds per water molecule as a function of the distance from the membrane center A monotonic increase is observed below 2 nm, where water molecules penetrate the bilayer and are surrounded by lipid headgroups. In this region, although more hydrogen bonds are still formed with water molecules than with lipids, the difference between w–w and w–l hydrogen bonding is reduced by approximately 50% at a distance of 1 nm from the membrane center. Inset: average number of w-l HB as a function of temperature The black line represents an Arrhenius fit, yielding an activation energy of 1.37 $\pm$ 0.25 kJ/mol.


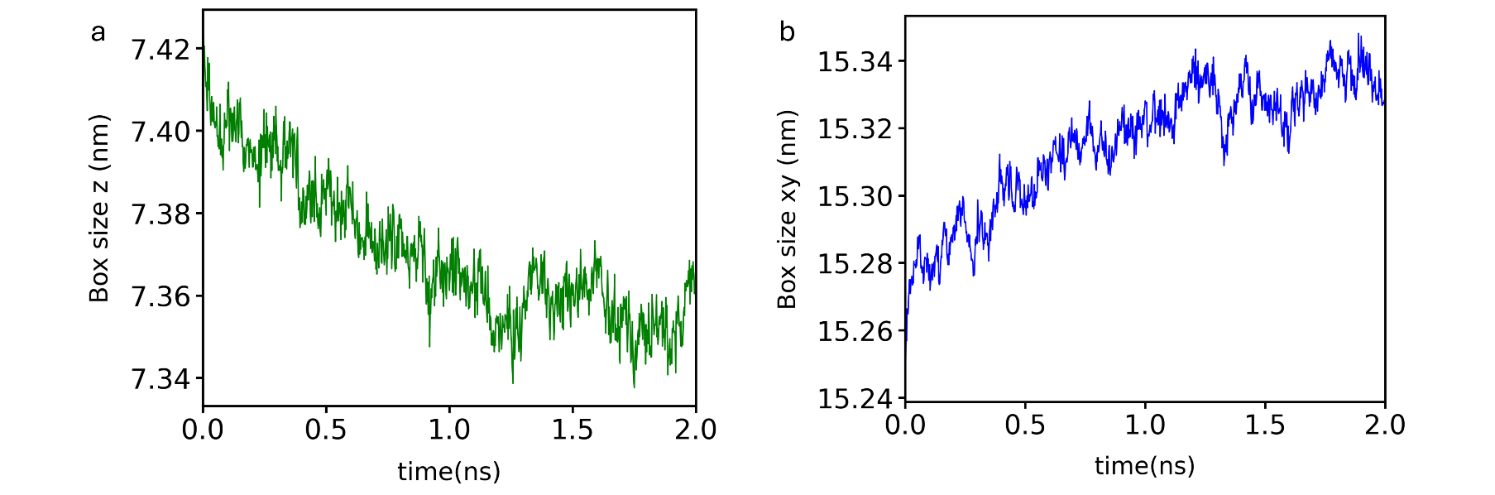


**Figure S9.** NPT equilibration. **a)** Size of the box in the z direction as a function of time. **b)** Size of the box in the xy direction, parallel to the water-lipid interface, as a function of time.


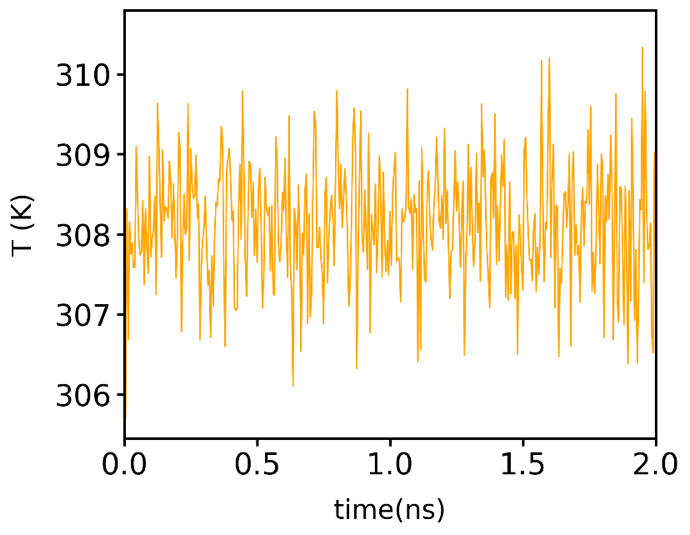


**Figure S10.** NVT equilibration. Temperature as a function of time.
